# Assessing Antiparasitic Compounds Persistence in Cattle Hair by DART-MS

**DOI:** 10.1021/jasms.4c00422

**Published:** 2024-11-27

**Authors:** Almir Custodio Batista Junior, Lanaia Ítala Louzeiro Maciel, Yuri Arrates Rocha, Gabriela Guimarães Souza, Boniek Gontijo Vaz, Welber Daniel Zanetti Lopes, Ana Flávia Machado Botelho, Marc Yves Chalom, Andréa Rodrigues Chaves

**Affiliations:** † Universidade Federal de Goiás, Instituto de Química, Goiânia, Goiás 74690-900, Brazil; ‡ Universidade Federal de Goiás, Escola de Veterinária e Zootecnia, Goiânia, Goiás 74690-900, Brazil; § SENS Advanced Mass Spectrometry, 05319-000, São Paulo, SP Brazil

**Keywords:** ambient ionization mass spectrometry, direct analysis, fenthion, chlorpyrifos, cypermethrin

## Abstract

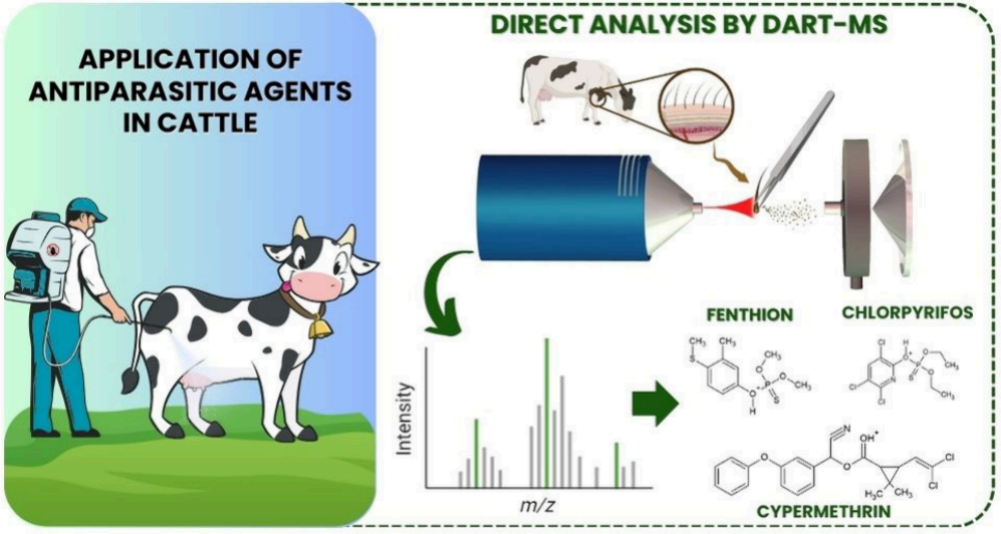

This study introduces an alternative strategy for evaluating antiparasitic persistence compounds in cattle hair by Direct Analysis in Real Time Mass Spectrometry (DART-MS). The developed DART-MS method aimed to determine fenthion, chlorpyrifos, and cypermethrin in cattle hair samples. DART-MS analyses were performed in positive ion mode, and parameters related to the DART source were evaluated. The analytical performance demonstrated the efficiency of the optimized DART-MS method for fenthion, chlorpyrifos, and cypermethrin quantification in the evaluated samples, meeting criteria for precision, accuracy and limits of detection. Overall, the DART-MS method provided a fast and efficient analysis for determination of antiparasitic agents in cattle hair, which contributes to the evaluation of drug administration protocols and dosage intervals, and aids the safety and advancement of the livestock sector.

## 1 Introduction

The livestock farming plays a significant role in Brazil’s economy, representing around 30% of the country’s GDP.^1,2^ Among the challenges faced in this sector, tick control is particularly pressing, as these parasites can jeopardize cattle health by facilitating the transmission of pathogenic agents, which limits productivity and leads to economic losses for farmers.^3−5^ In this regard, tick control in cattle requires the use of antiparasitic methods, and the main strategy involves the application of chemical agents.^3,6^

A wide range of antiparasitic agents such as fenthion, chlorpyrifos, and cypermethrin have gained attention due to their outstanding performance in tick control.^3,5^ Normally, all animals in the herd are treated with the antiparasitic agents at predetermined intervals, considering the tick’s biology, ecology, and the product’s residual effect.^7,8^ Furthermore, the control of ticks by chemicals has become increasingly challenging, with reports of tick-resistant populations rising significantly over the past years. Thus, the application of antiparasitic chemicals must be carefully managed to ensure an effective application and persistence in cattle hair.^7−9^

To determine the interval of application of antiparasitic compounds by its persistence in cattle hair, it is necessary to employ sensitive and robust analytical techniques. The literature presents several methods for the determination of fenthion, chlorpyrifos, and cypermethrin based on chromatography techniques coupled to mass spectrometry (MS).^10,11^ These conventional protocols may present drawbacks such as prolonged analysis time, time-consuming sample preparation, and higher solvents and sample volume. These factors increase the analysis costs and impair large-scale analysis, which is not desirable according to sustainable principles advocated by green analytical chemistry.^12,13^ In this scenario, ambient MS techniques, such as direct analysis in real-time mass spectrometry (DART-MS), emerge as alternative methodologies that allow direct analysis and eliminate the need for extensive sample preparation steps, reducing the duration of analysis, solvent, and residue volumes.^14^ DART-MS provides a rapid and efficient ionization method for MS, capable of measuring a wide range of analytes in their native forms.^15,16^

Given the considerations stated above, there is plenty of evidence that the DART-MS technique represents an alternative method for the evaluation of antiparasitic agents. Thus, the present study aimed to develop a reliable method for fenthion, chlorpyrifos, and cypermethrin screening and assessment in cattle hair. So far, the literature does not present the use of the DART-MS technique for antiparasitic agents’ evaluation, and this inauguration methodology represents new frontiers in the antiparasitic solution evaluation for livestock management.

## 2 Experimental Section

### 2.1 Chemicals and Reagents

Methanol (>99.90%) was acquired by Tedia (Ohio, USA). A mix surrogate solution of fenthion (187.5 mg L^–1^), chlorpyrifos (375 mg L^–1^), and cypermethrin (187.5 mg L^–1^) and pyrilamine (>99.90%) was acquired from Sigma-Aldrich (Pennsylvania, USA).

### 2.2 Samples

Cattle hair samples were acquired in collaboration with the Laboratory of Parasitological Specialties (LEPAR), Institute of Tropical Pathology and Public Health (IPTSP), and School of Veterinary and Zootechnics (EVZ) of the Universidade Federal de Goiás (UFG). This study is approved by the Animal Use Ethics Committee (CEUA protocol no 010/24).

Animals were treated with a commercial formulation containing fenthion, chlorpyrifos, and cypermethrin (Colosso FC 30 – Ouro Fino Animal Health). The solution was applied with a motorized stationary sprayer model (HS-22 Yamaha), operating with a pressure of 300 psi. The application pressure should be sufficient to ensure effective deposition and penetration of the formulation, which can play a critical role in maintaining the antiparasitic agents on the animal for several days.

The commercial product was diluted according to the manufacturer’s recommendations. The solution was applied using a hand pump with pressure in a full cone nozzle. Each animal was sprayed for approximately 5 min with 5 L of the solution, treating the entire body of each animal uniformly. Hair collection from each animal was carried out at moments 0 (before treatment), 6 h post-treatment, and daily for a period of 7 days post-treatment, with the aid of scissors. Approximately 1 g of hair was collected from each animal, from its dorsal region. The hair from each animal was stored in hermetic plastic bags and frozen (−20 °C) until the moment of evaluation ^9^.

For rapid screening of fenthion, chlorpyrifos, and cypermethrin, the cattle hair samples were analyzed directly by DART-MS with no sample preparation. For comparison purposes, the antiparasitic agents were also extracted from the cattle hair samples. Therefore, the extracts were obtained by adding 300 μL of methanol in 10 mg of cattle hair, adding pyrilamine (1.0 μg mL^–1^) as internal standard (IS) and this solution was vortexed for 1 min and the supernatant was separated and filtered for further analysis.

### 2.3 DART-MS Analysis

**Figure 1:**
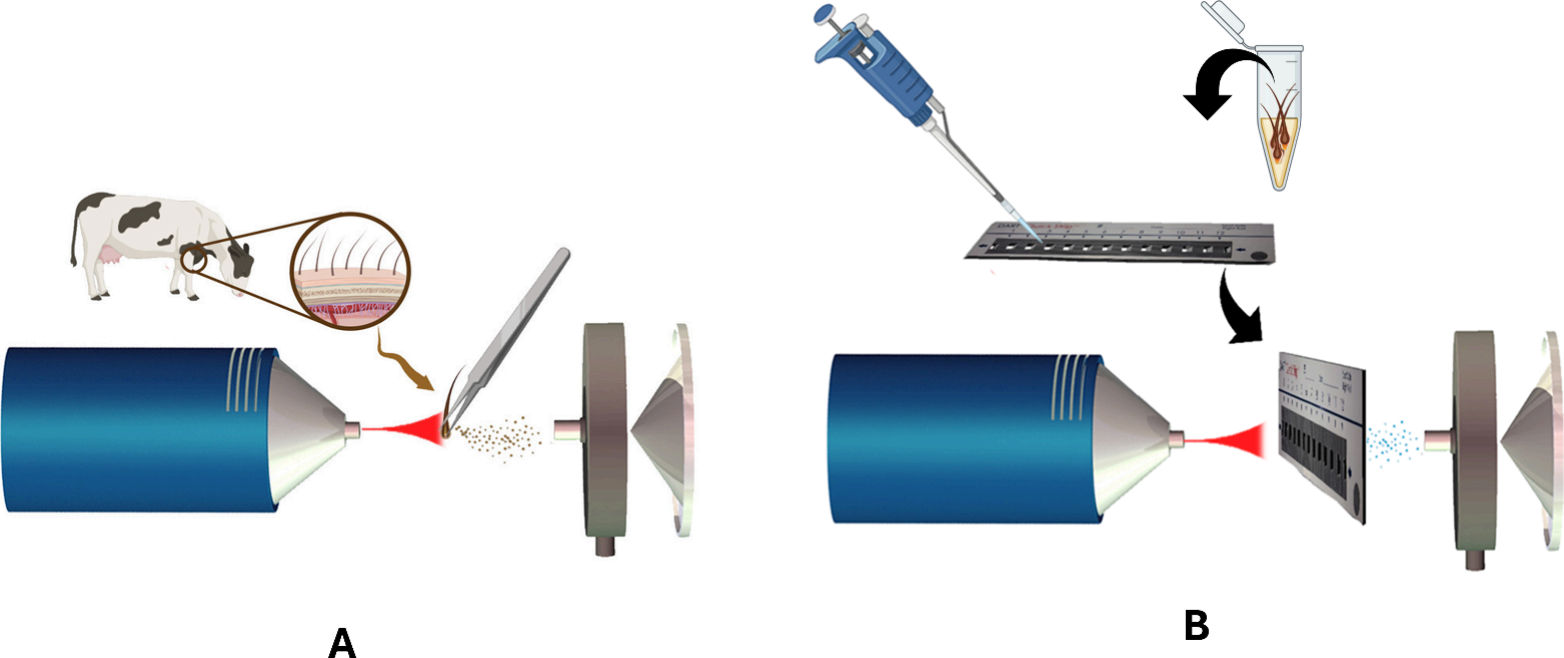
Representation of analysis of cattle hair by DART-MS. (A) *In situ* analysis of cattle hair. (B) Analysis of antiparasitic agents extracted from cattle hair using a QuickStrip card.

The antiparasitic agents in cattle hair samples were evaluated *in situ* and in extracts obtained from cattle hair via DART-MS (Figure 1). The analyses were conducted on a DART-VSP ion source (IonSense, Massachusetts, USA) coupled to a micrOTOF-Q III mass spectrometer (Bruker Daltonics, Bremen, Germany), operating in positive ion mode. DART ion source operated using helium (White Martins, 99.999% purity) as ionization gas, and its temperature was evaluated ranging from 100 to 300 °C, applying a grid voltage of 350 V and gas flow rate of 3.0 L min^–1^. The DART source was positioned at a distance of 30 mm from the mass spectrometer inlet for both *in situ* analysis and extract analysis. MS analyses accounted for a mass range of *m*/*z* 200–500 with an acquisition time of 30 s. Peak annotations for the antiparasitic agents considered the exact mass with a mass error lower than 25.0 ppm. MS/MS experiments were also conducted by DART-MS, applying collision energy ranging from 10–35 eV. The mass spectra were processed using DataAnalysis software version 5.0 (Bruker Daltonics, Bremen, Germany).

For *in situ* analysis of cattle hair, the hair samples were centrally positioned between the mass spectrometer inlet and the DART ion source with the assistance of metal tweezers (Figure 1A). As for the antiparasitic agent’s extract analysis, the analyses were carried out by adding 5 μL of the extract obtained from the cattle hair onto a QuickStrip template card (Figure S1) which was placed in a QuickStrip holder, placed between the DART ion source and the mass spectrometer inlet (Figure 1 B).

### 2.4 ESI-MS

Electrospray ionization mass spectrometry (ESI-MS) analyses were performed in a mass spectrometer micrOTOF-Q III (Bruker, Bremen, Germany), using an ESI source with the following key parameters: capillary temperature set to 180 °C, spray voltage at 4.5 kV, gas flow at 4 L min^–1^, and nebulizer pressure at 0.4 bar. Mass spectra were acquired in a *m*/*z* range of 200–500 in positive ion mode with an acquisition time of 1 min. The mass spectra were processed using DataAnalysis software version 5.0 (Bruker Daltonics, Bremen, Germany).

### 2.5 DART-MS Analytical Performance

The analytical performance of the DART-MS method for determination of fenthion, chlorpyrifos, and cypermethrin (Table 1) was evaluated according to the validation of analytical procedure guidelines prescribed by Brazilian guidelines based on ANVISA (National Health Surveillance Agency).^17^

**Table 1:**
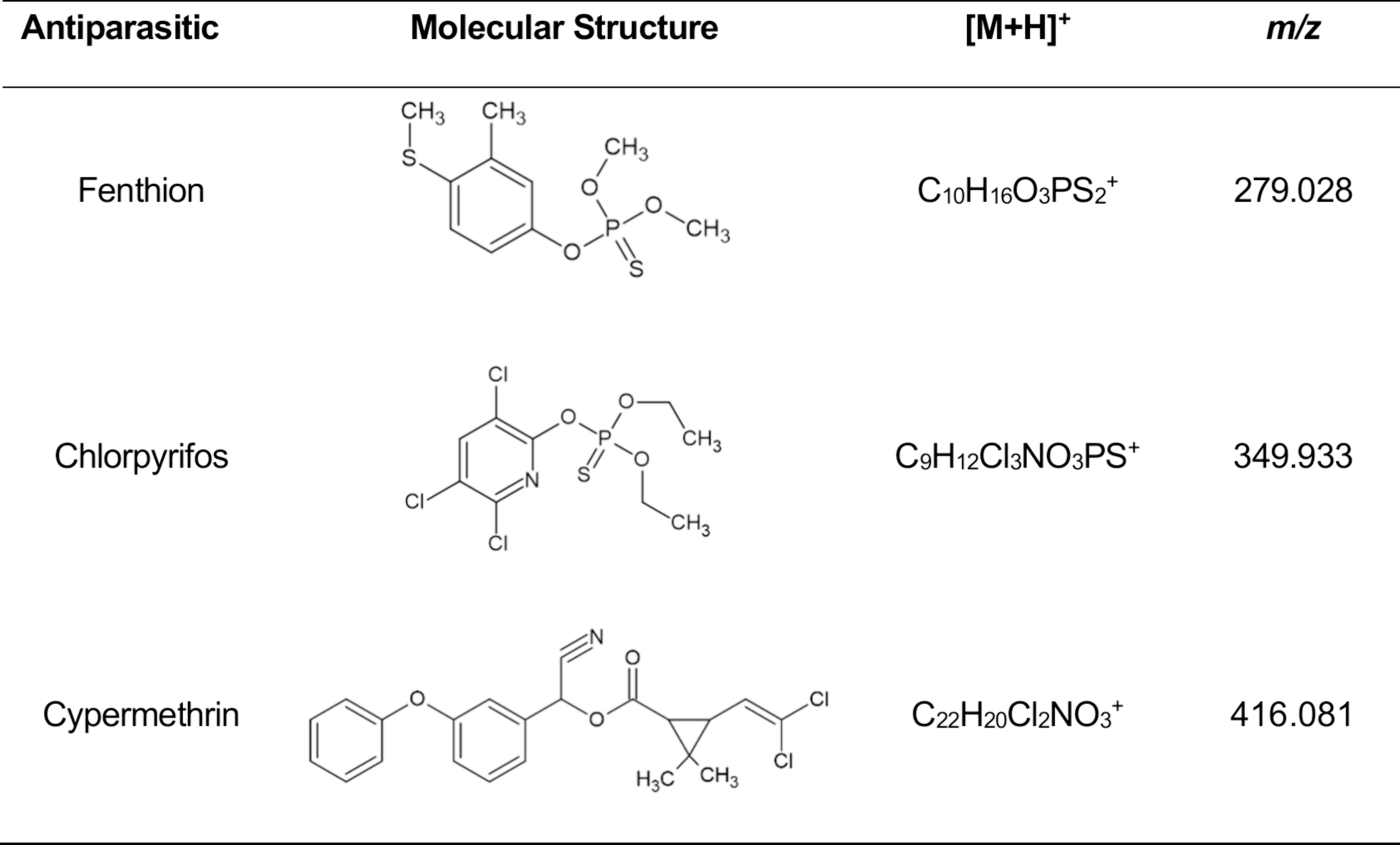
Antiparasitic Agents Used for the Method Development

Cattle hair of animals that had not been treated with antiparasitic agents was used as a control sample. Ten mg of the control hair was spiked with chlorpyrifos ranging from 0.050–10.0 ng mL^–1^ and fenthion and cypermethrin ranging from 0.025–5.0 ng mL^–1^, adding pyrilamine at 1.0 μg mL^–1^ as IS, in replicates (n = 5). The ratio of the analyte peak intensity to that of the IS was used as the relative analytical response and the linearity over the studied concentration range was evaluated using an analytical curve. The limit of detection (LOD) and limit of quantification (LOQ) for each analyte were determined based on the signal/noise ratio of 3 for LOD and 10 for LOQ. The matrix effect (ME%) was evaluated at three concentration levels of the analytes (0.5, 5.0, and 10.0 ng mL^–1^ for chlorpyrifos and 0.25, 2.0, and 5.0 ng mL^–1^ for fenthion and cypermethrin) according to eq 1 where AM represents the analytical response for the analyte in the matrix, and AS represents the analytical response of the analyte in the solution. Precision (P%) and accuracy (A%) were calculated based on eqs 2 and 3, respectively, where SD is the standard deviation of the experimental concentration, AEC is the average experimental concentration and TC is the theoretical concentration.
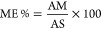
1
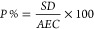
2
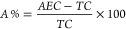
3

### 2.6 Determination of Antiparasitic Agents in Real Samples

Cattle hair samples were collected over 7 days to investigate the persistence of fenthion, chlorpyrifos, and cypermethrin in the animals using the DART-MS method. Extracts were prepared by adding 300 μL of methanol to 10 mg of cattle hair, with IS (1.0 μg mL^–1^). The mixture was vortexed for 1 min, and the supernatant was then separated and filtered for DART-MS analysis.

## 3 Results and Discussion

### 3.1 DART-MS Method Development

The DART-MS technique was used to evaluate antiparasitic compounds in cattle hair. This method was developed to identify and quantify fenthion, chlorpyrifos, and cypermethrin, both through *in situ* analysis and through the analysis of extracts from cattle hair samples. The technique was specifically optimized to detect these compounds in cattle hair. Recent studies conducted by our research group have demonstrated the importance of optimizing the parameters of the DART ion source, such as the choice of ionization gas (N_2_ or He), the distance from the DART source to the mass spectrometer inlet, and the temperature of the ionization gas in its metastable form.^18^

Regarding the ionization gas, the literature has demonstrated that helium (He) is more efficient in ionizing compounds than nitrogen (N_2_),^19,20^ a finding corroborated by the analysis of cattle hair. In positive ion mode, DART ionizes compounds following a Penning ionization mechanism which is the first step of reactions that leads to the formation of H_3_O^+^ species.^15^ The superior ionization efficiency of He arises from its higher internal energy in its metastable state, compared to N_2_, improving the formation of charged clusters and favoring the ionization mechanism.^19,20^ Therefore, He was used for further analysis of cattle hair.

Another important parameter related to the DART-MS method is the distance between the DART ion source and the mass spectrometer inlet. This distance allows for direct and effective interaction between the metastable gas and the sample. For *in situ* analysis of cattle hair samples, maintaining an appropriate distance is important not only to ensure adequate sample ionization but also to avoid the sample from entering the mass spectrometer. Literature indicates that a distance around 10–25 mm between the DART ion source and the mass spectrometer inlet optimizes gas-sample interaction, yielding great signal intensity.^21^ However, considering the safety of the mass spectrometer and the intensity of the analytes in mass spectra, a distance of 30 mm was selected, which prevented the entry of particulate matter into the equipment and generated an adequate analytical response.

The temperature of the ionization gas is intrinsically related to the compounds assessed by the DART source, as an increase in the gas temperature can induce the thermal desorption of a great number of compounds from the matrix, including those less volatile compounds. Therefore, tests were conducted regarding the gas temperature ranging from 100 to 300 °C using the cattle hair samples *in situ* (Figure S2). Through Figure S2, it was possible to observe that fenthion (*m*/*z* 279.028) and chlorpyrifos (*m*/*z* 349.933) demonstrated greater analytical response (intensity of the analytes) in the spectra obtained at temperatures of 150 and 200 °C. As for cypermethrin (*m*/*z* 416.081), the best results were obtained at temperatures above 200 °C. In addition to evaluating these antiparasitic agents, it is necessary to consider the stability of the sample with the temperature. Thermal analysis studies indicate that hair is thermally stable up to 224 °C, beyond which degradation occurs.^22^ Based on these findings, further analyses were conducted at 200 °C for both *in situ* and extract analysis aiming to keep the same parameters for results comparing purposes. This temperature allows for the assessment of fenthion, chlorpyrifos, and cypermethrin without compromising the sample integrity.

**Figure 2:**
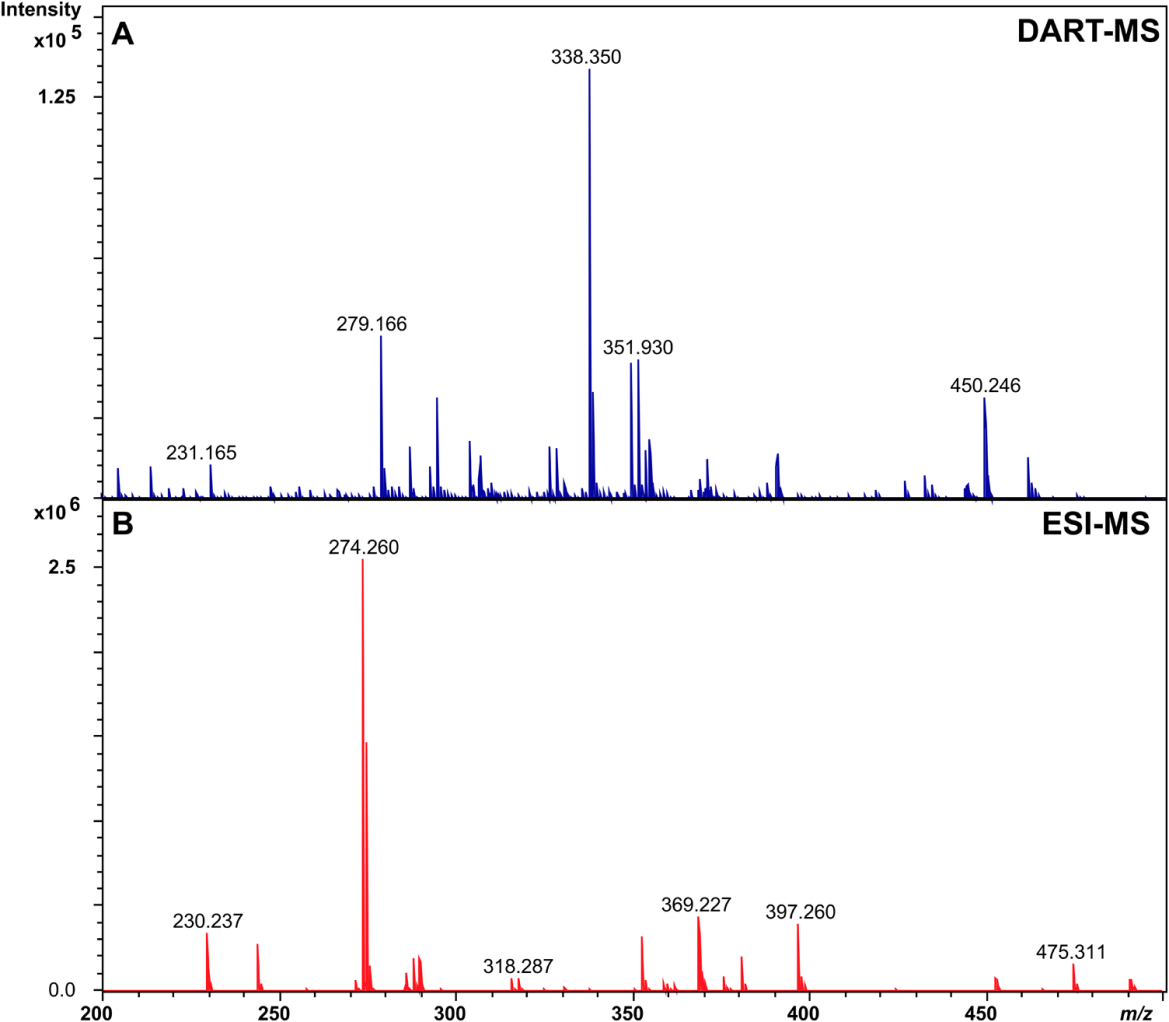
Mass spectra obtained from the analysis of cattle hair. (A) Spectrum acquired by DART-MS from the analysis of cattle hair *in situ*. (B) Spectrum acquired by direct infusion using ESI-MS in cattle hair extracts.

ESI-MS experiments were conducted to evaluate the ion profile of cattle hair extracts in comparison with *in situ* DART-MS analysis operating with He as ionization gas in temperature of 200 °C. As shown in Figure 2A and Figure 2B, a clear visual difference between the mass spectra obtained via direct infusion using ESI-MS and DART-MS is evident within the mass range from *m*/*z* 200–500. The mass spectra from ESI-MS exhibited lower sensitivity compared to those from DART-MS.

Additionally, Figure 2A and Figure 2B highlight differences in intensity between the two methods, with DART-MS providing a stronger and more consistent signal, indicating superior performance in the analysis of these samples. This difference may be attributed to the efficiency of each technique with compounds of different polarities and its mechanisms of ionization. DART-MS provides more efficient ionization for small and nonpolar compounds, resulting in higher sensitivity, while ESI-MS efficiently ionizes a broader range of polar molecules. Moreover, the selectivity of the DART technique relies on the requirement for a certain degree of volatility in the evaluated molecules, as ionization occurs through thermal desorption of the analytes, followed by a Penning ionization mechanism.^15^ Thus, the gas temperature plays a fundamental role in the ionization of low-volatility molecules, as previously detailed.

Also, the difference in the analytical signal for fenthion, chlorpyrifos, and cypermethrin comparing ESI and DART ion sources is evident (Figure S3). The ionization of these compounds was notably enhanced when using the DART source at a gas temperature of 200 °C, as this temperature promoted their thermal desorption and minimized the ionization of matrix-related interferences, a challenge that is often difficult to overcome with ESI-MS. Therefore, it is evident that ESI-MS requires sample preparation prior to analysis for clean-up and preconcentration of the analytes, unlike DART-MS, where the analysis occurs *in situ* without sample preparation.

### 3.2 DART-MS Analytical Performance

For the evaluation of the analytical performance of the developed method, samples of cattle hair that had not been treated with the target compounds were used as the reference blank samples. Extracts were prepared, spiking the cattle hair with fenthion (0.025–5.0 ng mL^–1^), chlorpyrifos (0.050–10.0 ng mL^–1^) and cypermethrin (0.025–5.0 ng mL^–1^). Table 2 presents the values obtained through the regressions, which demonstrated great linearity for the three analytes in the concentration range studied (r^2^ > 0.99). LOD and LOQ, also presented in Table 2, were determined based on the signal/noise ratio obtained experimentally.

**Table 2:** Linear Regression, LOD and LOQ Values for the Antiparasitic Agents Evaluated by the DART-MS Method

Analyte	Linear regression	r^2^	LOD (ng mL^–1^)	LOQ (ng mL^–1^)
Fenthion	y = 0.0171x + 0.0020	0.995	0.010	0.025
Chlorpyrifos	y = 0.0137x + 0.0026	0.996	0.010	0.050
Cypermethrin	y = 0.0012x + 0.00005	0.993	0.010	0.025

The matrix effect was assessed at three concentration levels (low, medium and high) for the antiparasitic agents analyzed by DART-MS. This effect was determined based on the analytical response (analyte/IS) of the analytes in the cattle hair matrix compared to the analytes in the absence of the matrix (methanol), as described by eq 1. According to this equation, the matrix effect is not considered significant for values between 80% and 120%. The results, presented in Table 3, indicate that fenthion, chlorpyrifos, and cypermethrin did not exhibit a significant matrix effect, with calculated values ranging from 85% to 117%, thereby complying with the stipulated criteria.^17^ Precision and accuracy were assessed at three different concentration levels for each analyte, calculated using eqs 2 and 3, respectively. The resulting values for these figures of merit for DART-MS method were below 12%, complying with regulatory guidelines that set a limit of 15%.^17^ Precision and accuracy were assessed at three different concentration levels for each analyte, calculated using eqs 2 and 3, respectively. The resulting values for these figures of merit for DART-MS method were below 12%, complying with regulatory guidelines that set a limit of 15%.^17^

**Table 3:** Precision, Accuracy, and Matrix Effect Values for the Analysis of the Antiparasitic Agents by DART-MS

Analyte	Concentration (ng mL^–1^)	Precision %	Accuracy %	Matrix Effect %
Fenthion	0.25	5.0	–0.5	89.2
	2.5	1.7	6.4	98.2
	5.0	1.4	–2.0	112.8
Chlorpyrifos	0.50	8.5	12.7	85.0
	5.0	4.6	7.9	108.0
	10.0	3.2	–1.8	117.8
Cypermethrin	0.25	10.4	–12.4	92.4
	2.5	9.9	–13.4	92.3
	5.0	14.3	2.6	85.5

The literature describes various methods for determination of fenthion, chlorpyrifos and cypermethrin in different matrices. Most of these methods are based on liquid chromatography or gas chromatography coupled to mass spectrometry. These techniques demonstrate robustness and sensitivity for evaluation of these antiparasitic agents in various matrices, such as food samples and plasma samples, showing linearity and appropriate LOD and LOQ values for the analytes.^23−25^ However, these methods have drawbacks, including the requirement for large amounts of solvent and extended analysis time, as well as the need for a preliminary step for sample cleaning and preconcentration before analysis. These factors hinder large-scale analyses and are not in line with more sustainable analytical methods.

Conversely, the DART-MS technique offers significant advantages in terms of analysis time, solvent consumption, and the potential for *in situ* analyses. The developed method requires only 30 s per assay, demonstrating readiness for high-throughput and facilitating large scale analysis. Additionally, it shows excellent analytical performance in the simultaneous determination of fenthion, chlorpyrifos, and cypermethrin, commonly coapplied antiparasitic agents. DART-MS analysis delivers a rapid and effective analytical response, with suitable LOQ and LOD, as well as appropriate precision and accuracy values.

### 3.3 In Situ Analysis through DART-MS

**Figure 3:**
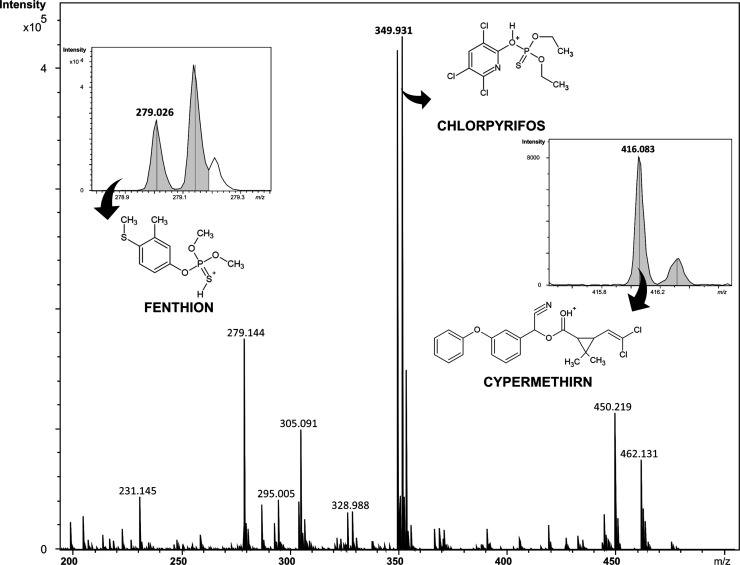
Mass spectra obtained for the *in situ* cattle hair analysis obtained by the developed method using DART-MS.

The presence of fenthion, chlorpyrifos and cypermethrin in cattle hair was evaluated using the developed DART-MS method for *in situ* analysis. *In situ* analyses were carried out positioning the cattle hair between the DART ion source and the mass spectrometer inlet. Figure 3 demonstrates the mass spectra obtained by *in situ* analysis using the optimized parameters (He as ionization gas, distance of 30 mm and gas temperature of 200 °C). Fenthion (*m*/*z* 279.026, mass error = 7.1), chlorpyrifos (*m*/*z* 349.931, mass error = 5.7), and cypermethrin (*m*/*z* 416.083, mass error = 4.8) were detected by this approach, exhibiting a mass error of less than 10 ppm. The analytes were confirmed by MS/MS experiments (Figure S4 and S5). Fenthion exhibited a strong signal from a fragment ion at *m*/*z* 247.005, corresponding to an intramolecular rearrangement with the loss of 32.007 Da, attributed to a methanol molecule (−CH_3_OH) (see details in Figure S4), consistent with previously reported studies.^26^ In the case of chlorpyrifos, an intense signal from a fragment ion at *m*/*z* 321.927 was observed, corresponding to the loss of 28.005 Da (C_2_H_4_) (Figure S5) which is also consistent with the literature.^27^ It was not possible to obtain a fragmentation profile for cypermethrin due to its instability observed during MS/MS experiments. However, the low mass error strongly suggests its presence.

This approach demonstrated a rapid and feasible way to evaluate these antiparasitic agents in cattle hair without any sample preparation. Despite its advantages, *in situ* analysis was unable to quantify these analytes in cattle hair samples due to the absence of an internal standard.

### 3.4 Determination of Fenthion, Chlorpyrifos, and Cypermethrin in Cattle Hair

To evaluate the persistence of antiparasitic agents in real samples, cattle hair samples were collected on the day of application of the antiparasitic agent containing fenthion, chlorpyrifos, and cypermethrin, as well as on the following 7 days. Thus, hair samples were collected over 8 days from the same location on the animal (dorsal area). The analytes were extracted from these samples, and the extracts were analyzed by DART-MS.

**Figure 4:**
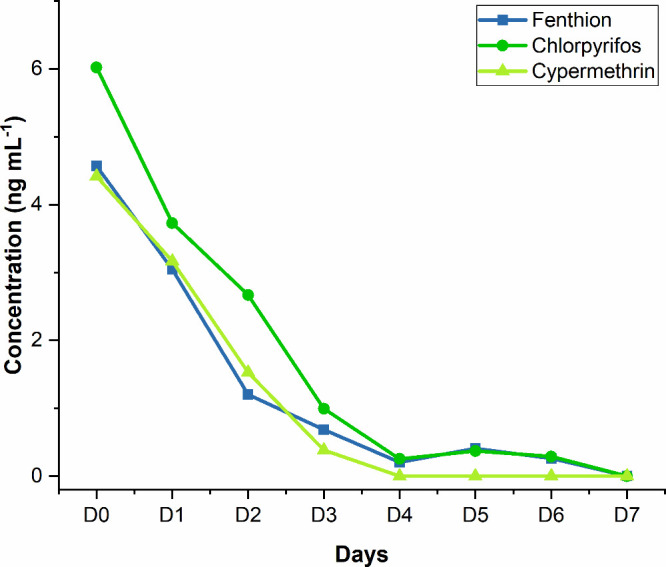
Persistence of fenthion, chlorpyrifos, and cypermethrin in cattle dorsal hair over days.

Fenthion, chlorpyrifos, and cypermethrin were quantified in these samples, according to the linear regression (Table 2), and the results are displayed in Figure 4. The DART-MS method was able to quantify these compounds at trace levels (ng mL^–1^). The decay behavior of these compounds, analyzed using linear regression, appears to follow first-order kinetics, where a rapid decline in concentration occurs initially, followed by a more gradual reduction as the levels approach trace amounts. This kinetic profile is typical of the environmental degradation of pesticides and antiparasitic agents, which initially degrade quickly due to higher concentrations, and then decay more slowly as the residue levels decrease.

The DART-MS method was able to monitor the temporal degradation of compounds, offering real-time analysis without extensive sample preparation. Its ability to detect low concentrations of chemical residues over time makes it valuable for rapid environmental monitoring. This highlights its utility in accurately assessing the persistence of antiparasitic agents and their potential impacts on animal health and environmental safety.

This type of evaluation allows the monitoring of the persistence of these compounds in cattle hair is essential for the eradication of parasites, such as ticks and improve the producers’ practice by reducing the improper formulations use. Using this information on the persistence of these compounds in cattle hair, decisions can be made regarding the optimal application concentration, spray pressure, and the interval of application. Thus, the DART-MS technique proves to be a valuable tool in veterinary studies, enabling evaluations and contributing to the safe use of these antiparasitic agents.

### 3.5 Green Aspects

Finally, the sustainability of the DART-MS method for determining antiparasitic compounds and its adherence to green analytical chemistry principles, the AGREE^28^ metric was employed (Figure S6). This metric considers the 12 green analytical chemistry principles and converts them into scores. The AGREE result for the developed method is illustrated in Figure S6, using a pictogram that displays the overall score of 0.77, which highlights its sustainability and greenness

## 4 Conclusions

DART-MS is a well-established technique widely used in various analytical fields, including the determination of trace-level analytes. The DART-MS method provided a fast and effective analysis of fenthion, chlorpyrifos and cypermethrin in cattle hair samples. *In situ* analysis of cattle hair for these antiparasitic agents proved suitable for rapid screening without any sample preparation. For quantification, the method demonstrated precision and accuracy below 15%, suitable LOD and LOQ values and no significant matrix effect. The AGREE metric further validates the excellence of DART-MS by attending some criteria postulated by the green analytical chemistry, which demonstrates its sustainability. Therefore, the method developed using DART-MS technique represents a valuable asset to the livestock sector by providing a rapid and efficient means of detecting the persistence of antiparasitic agents in animals. This method can serve as a reference for evaluating drug administration protocols and dosage intervals, thereby enhancing both the safety and development of the sector.

## Supporting Information Available

QuickStrip card; evaluation of the antiparasitic agents regarding the gas temperature; comparison between DART and ESI ion source regarding the antiparasitic compounds; MS/MS spectra for the three analytes; AGREE metric for the DART-MS method (PDF)

## References

[ref1] de Assis D. C. S.; da Silva T. M. L.; Brito R. F.; da Silva L. C. G.; Lima W. G.; Brito J. C. M. Shiga Toxin-Producing Escherichia Coli (STEC) in Bovine Meat and Meat Products over the Last 15 Years in Brazil: A Systematic Review and Meta-Analysis. Meat Sci. 2021, 173, 108394 10.1016/j.meatsci.2020.108394. 33316706

[ref2] Viçoso L. C. B. A Pecuária Como Agente de Territorialização e as Formas de Fomento Para Sustentação Da Pecuária. CARDERNOS DO LESTE 2021, 21 (21), 1 10.29327/248949.21.21-6.

[ref3] Ferré D. M.; Ludueña H. R.; Romano R. R.; Gorla N. B. M. Evaluation of the Genotoxic Potential of Cypermethrin, Chlorpyrifos and Their Subsequent Mixture, on Cultured Bovine Lymphocytes. Chemosphere 2020, 243, 125341 10.1016/j.chemosphere.2019.125341. 31751924

[ref4] Hennessey M.; Whatford L.; Payne-Gifford S.; Johnson K. F.; Van Winden S.; Barling D.; Häsler B. Antimicrobial & Antiparasitic Use and Resistance in British Sheep and Cattle: A Systematic Review. Prev Vet Med. 2020, 185, 105174 10.1016/j.prevetmed.2020.105174. 33189057

[ref5] Nicaretta J. E.; Couto L. F. M.; Heller L. M.; Ferreira L. L.; Cavalcante A. S. de A.; Zapa D. M. B.; Cruvinel L. B.; Júnior R. D. de M.; Gontijo L. M. de A.; Soares V. E.; Mello I. A. S.; Monteiro C. M. de O.; Lopes W. D. Z. Evaluation of Different Strategic Control Protocols for Rhipicephalus Microplus on Cattle According to Tick Burden. Ticks Tick Borne Dis 2021, 12 (4), 101737 10.1016/j.ttbdis.2021.101737. 33984596

[ref6] Githaka N. W.; Kanduma E. G.; Wieland B.; Darghouth M. A.; Bishop R. P. Acaricide Resistance in Livestock Ticks Infesting Cattle in Africa: Current Status and Potential Mitigation Strategies. Current Research in Parasitology & Vector-Borne Diseases 2022, 2, 100090 10.1016/j.crpvbd.2022.100090. 35664895 PMC9160480

[ref7] Reckziegel G. H.; de Freitas M. G.; Tutija J. F.; Rodrigues V. D.; Borges D. G. L.; de Freitas M. D. B.; Gallina T.; Lopes W. D. Z.; de Castro Rodrigues D.; de Oliveira Arriero Amaral H.; Strydom T.; Torres S.; de Almeida Borges F. Efficiency of Fluralaner Pour-on in Different Strategic Control Protocols against Rhipicephalus Microplus on Brangus Cattle in a Tropical Area. Parasit Vectors 2024, 17 (1), 110 10.1186/s13071-024-06199-4. 38449052 PMC10916271

[ref8] Nicaretta J. E.; de Melo Junior R. D.; Naves R. B.; de Morais I. M. L.; Salvador V. F.; Leal L. L. L. L.; Teixeira A. L. C.; Ferreira L. L.; Klafke G. M.; Monteiro C. M. de O.; Borges F. de A.; Costa Junior L. M.; Rodrigues D. S.; Lopes W. D. Z. Selective versus Strategic Control against Rhipicephalus Microplus in Cattle: A Comparative Analysis of Efficacy, Animal Health, Productivity, Cost, and Resistance Management. Vet Parasitol 2023, 321, 109999 10.1016/j.vetpar.2023.109999. 37556962

[ref9] Moraes N.; Nicaretta J. E.; Rodrigues D. de C.; Gonzaga B. C. F.; Barrozo M. M.; Vale F. L.; Pereira e Sousa L. J.; Coutinho A. L.; Gomes G. W.; Teixeira W. F. P.; Lopes W. D. Z.; Monteiro C. Comparison of the Efficacy of Different Methods to Apply Acaricides for Control of Rhipicephalus (Boophilus) Microplus. Ticks Tick Borne Dis 2023, 14 (4), 102190 10.1016/j.ttbdis.2023.102190. 37167772

[ref10] Gao B.; Poma G.; Malarvannan G.; Dumitrascu C.; Bastiaensen M.; Wang M.; Covaci A. Development of an Analytical Method Based on Solid-Phase Extraction and LC-MS/MS for the Monitoring of Current-Use Pesticides and Their Metabolites in Human Urine. Journal of Environmental Sciences 2022, 111, 153–163. 10.1016/j.jes.2021.03.029. 34949345

[ref11] Tabasum H.; Neelagund S. E.; Kotresh K. R.; Gowtham M. D.; Sulochana N. Estimation of Chlorpyrifos Distribution in Forensic Visceral Samples and Body Fluids Using LCMS Method. J. Forensic Leg Med. 2022, 91, 102423 10.1016/j.jflm.2022.102423. 35995011

[ref12] Kannaiah K. P.; Sugumaran A.; Chanduluru H. K.; Rathinam S. Environmental Impact of Greenness Assessment Tools in Liquid Chromatography - A Review. Microchemical Journal 2021, 170, 106685 10.1016/j.microc.2021.106685.

[ref13] Sajid M.; Płotka-Wasylka J. Green Analytical Chemistry Metrics: A Review. Talanta 2022, 238, 123046 10.1016/j.talanta.2021.123046. 34801903

[ref14] Zacometti C.; Tata A.; Stella R.; Leone S.; Pallante I.; Merenda M.; Catania S.; Pozzato N.; Piro R. DART-HRMS Allows the Detection of Toxic Alkaloids in Animal Autopsy Specimens and Guides the Selection of Confirmatory Methods in Accidental Plant Poisoning. Anal. Chim. Acta 2023, 1264, 341309 10.1016/j.aca.2023.341309. 37230724

[ref15] Gross J. H. Direct Analysis in Real Time—a Critical Review on DART-MS. Anal Bioanal Chem. 2014, 406 (1), 63–80. 10.1007/s00216-013-7316-0. 24036523

[ref16] Malihi F.; Wang T. An Improved Analytical Method for Quantitation of Nitrosamine Impurities in Ophthalmic Solutions Using Liquid Chromatography with Tandem Mass Spectrometry. Journal of Chromatography Open 2022, 2, 100037 10.1016/j.jcoa.2022.100037.

[ref17] Agência Nacional de Vigilância Sanitária - ANVISA. RESOLUÇÃO DA DIRETORIA COLEGIADA - RDC N° 166, DE 24 DE JULHO DE 2017 - Dispõe Sobre a Validação de Métodos Analíticos e Dá Outras Providências; 2017.

[ref18] Batista Junior A. C.; Bernardo R. A.; Rocha Y. A.; Vaz B. G.; Chalom M. Y.; Jardim A. C.; Chaves A. R. An Agile and Accurate Approach for N-Nitrosamines Detection and Quantification in Medicines by DART-MS. J. Am. Soc. Mass Spectrom. 2024, 35, 1657 10.1021/jasms.4c00012. 38716699

[ref19] Hajslova J.; Cajka T.; Vaclavik L. Challenging Applications Offered by Direct Analysis in Real Time (DART) in Food-Quality and Safety Analysis. TrAC Trends in Analytical Chemistry 2011, 30 (2), 204–218. 10.1016/j.trac.2010.11.001.

[ref20] An S.; Liu S.; Cao J.; Lu S. Nitrogen-Activated Oxidation in Nitrogen Direct Analysis in Real Time Mass Spectrometry (DART-MS) and Rapid Detection of Explosives Using Thermal Desorption DART-MS. J. Am. Soc. Mass Spectrom. 2019, 30 (10), 2092–2100. 10.1007/s13361-019-02279-3. 31368004

[ref21] Dumlao M.; Khairallah G. N.; Donald W. A. Internal Energy Deposition in Dielectric Barrier Discharge Ionization Is Significantly Lower than in Direct Analysis in Real-Time Mass Spectrometry. Aust. J. Chem. 2017, 70 (11), 1219 10.1071/CH17440.

[ref22] Brebu M.; Spiridon I. Thermal Degradation of Keratin Waste. J. Anal Appl. Pyrolysis 2011, 91 (2), 288–295. 10.1016/j.jaap.2011.03.003.

[ref23] Aydın Urucu O.; Beyler Çiğil A.; Birtane H.; Kök Yetimoğlu E.; Kahraman M. V. Selective Molecularly Imprinted Polymer for the Analysis of Chlorpyrifos in Water Samples. Journal of Industrial and Engineering Chemistry 2020, 87, 145–151. 10.1016/j.jiec.2020.03.025.

[ref24] Liao H.-T.; Hsieh C.-J.; Chiang S.-Y.; Lin M.-H.; Chen P.-C.; Wu K.-Y. Simultaneous Analysis of Chlorpyrifos and Cypermethrin in Cord Blood Plasma by Online Solid-Phase Extraction Coupled with Liquid Chromatography-Heated Electrospray Ionization Tandem Mass Spectrometry. Journal of Chromatography B 2011, 879 (21), 1961–1966. 10.1016/j.jchromb.2011.05.028. 21665556

[ref25] Lee J.; Kim J.-H. Simultaneous Analysis of Fenthion and Its Five Metabolites in Produce Using Ultra-High Performance Liquid Chromatography-Tandem Mass Spectrometry. Molecules 2020, 25 (8), 1938 10.3390/molecules25081938. 32331373 PMC7221716

[ref26] García-Vara M.; Postigo C.; Palma P.; Bleda M. J.; López de Alda M. QuEChERS-Based Analytical Methods Developed for LC-MS/MS Multiresidue Determination of Pesticides in Representative Crop Fatty Matrices: Olives and Sunflower Seeds. Food Chem. 2022, 386, 132558 10.1016/j.foodchem.2022.132558. 35339080

[ref27] Prata R.; López-Ruiz R.; Petrarca M. H.; Teixeira Godoy H.; Garrido Frenich A.; Romero-González R. Targeted and Non-Targeted Analysis of Pesticides and Aflatoxins in Baby Foods by Liquid Chromatography Coupled to Quadrupole Orbitrap Mass Spectrometry. Food Control 2022, 139, 109072 10.1016/j.foodcont.2022.109072.

[ref28] Pena-Pereira F.; Wojnowski W.; Tobiszewski M. AGREE—Analytical GREEnness Metric Approach and Software. Anal. Chem. 2020, 92 (14), 10076–10082. 10.1021/acs.analchem.0c01887. 32538619 PMC7588019

