# Supplementary material for: Assessing Antiparasitic Compounds Persistence in Cattle Hair by DART-MS
Source: J Am Soc Mass Spectrom. 2024 Nov 27;36(1):201–8. doi: 10.1021/jasms.4c00422 (PMC11697339; doi:10.1021/jasms.4c00422)
Supplement: Supplementary file 1 — js4c00422_si_001.pdf [file js4c00422_si_001.pdf]

ASSESSING ANTIPARASITIC COMPOUNDS PERSISTENCE IN CATTLE HAIR BY DART-MS

Almir Custodio Batista Junior [1], Lanaia Ítala Louzeiro Maciel[1], Yuri Arrates Rocha [1], Gabriela Guimarães Souza [1], Boniek Gontijo Vaz[1], Welber Daniel Zanetti Lopes[2], Ana Flávia Machado Botelho[2], Marc Yves Chalom[3], Andréa Rodrigues Chaves *[1]

[1] Universidade Federal de Goiás, Instituto de Química, Goiânia, Goiás 74690-900, Brazil

[2] Universidade Federal de Goiás, Escola de Veterinária e Zootecnia, Goiânia, Goiás 74690-900, Brazil

[3] SENS Advanced Mass Spectrometry, 05319-000, São Paulo, SP, Brazil

*andrea_chaves@ufg.br

Supporting Information

TABLE OF CONTENTS

TABLE OF CONTENTS		
**Figure S1**	QuickStrip™ template card used in the medicine solution analysis by DART-MS	**S-3**
**Figure S2**	Evaluation of fenthion (*m/z* 279.028), chlorpyrifos (*m/z* 349.933) and cypermethrin (*m/z* 416.081) in relation to the gas temperature variation	**S-3**
**Figure S3**	Comparison between DART and ESI ion source regarding the intensity of fenthion (*m/z* 279.028), chlorpyrifos (*m/z* 349.933) and cypermethrin (*m/z* 416.081)	**S-4**
**Figure S4**	MS/MS spectra for fenthion (*m/z* 279.028)	**S-4**
**Figure S5**	MS/MS spectra for chlorpyrifos (*m/z* 349.933)	**S-5**
**Figure S6**	AGREE metric for the determination of antiparasitic compounds in cattle hair by DART-MS	**S-5**

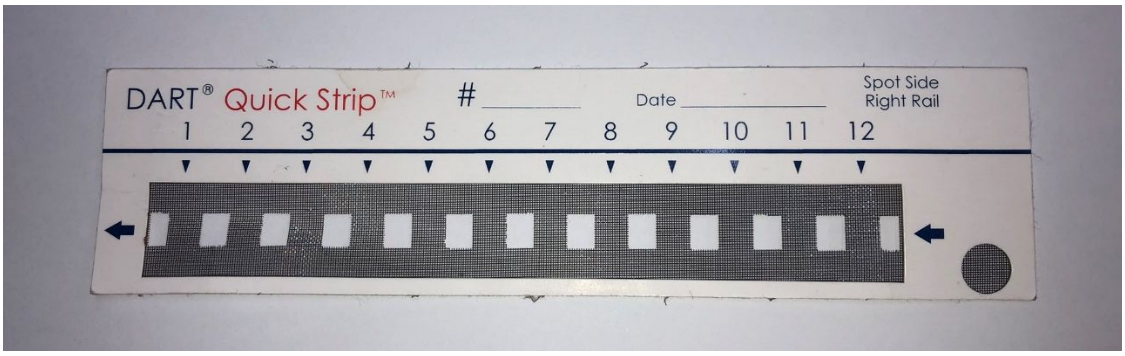

Figure S1: QuickStrip™ template card used in the medicine solution analysis by DART-MS

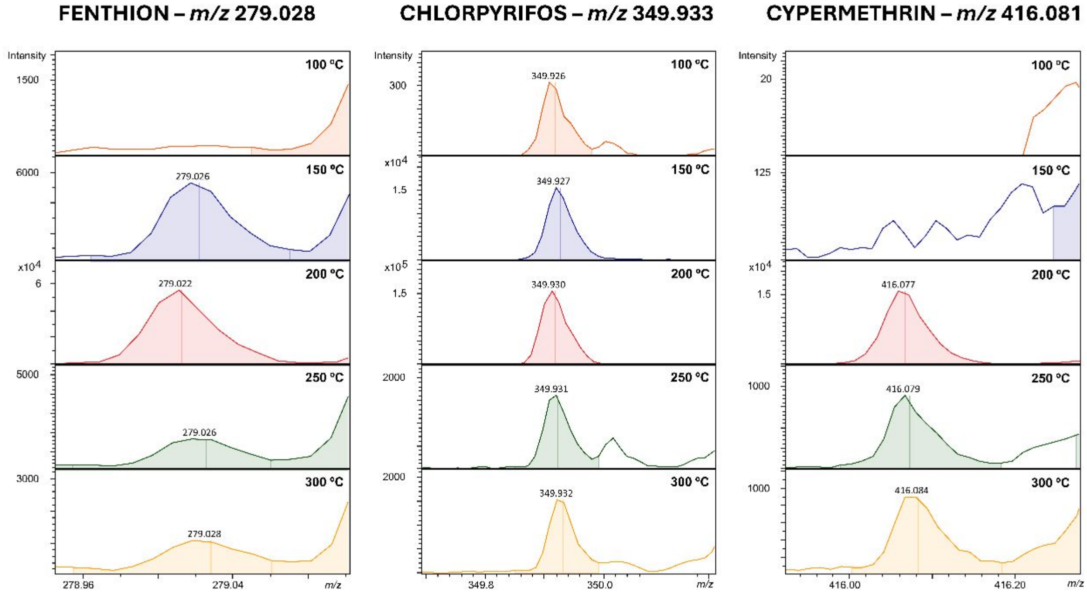

Figure S2: Evaluation of fenthion (*m/z* 279.028), chlorpyrifos (*m/z* 349.933) and cypermethrin (*m/z* 416.081) in relation to the gas temperature variation

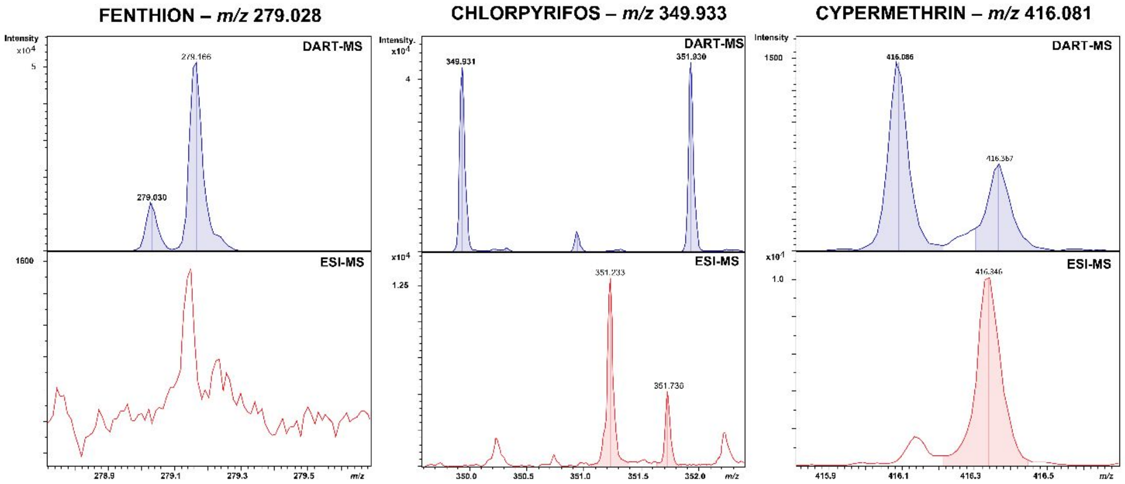

Figure S3: Comparison between DART and ESI ion source regarding the intensity of fenthion (*m/z* 279.028), chlorpyrifos (*m/z* 349.933) and cypermethrin (*m/z* 416.081)

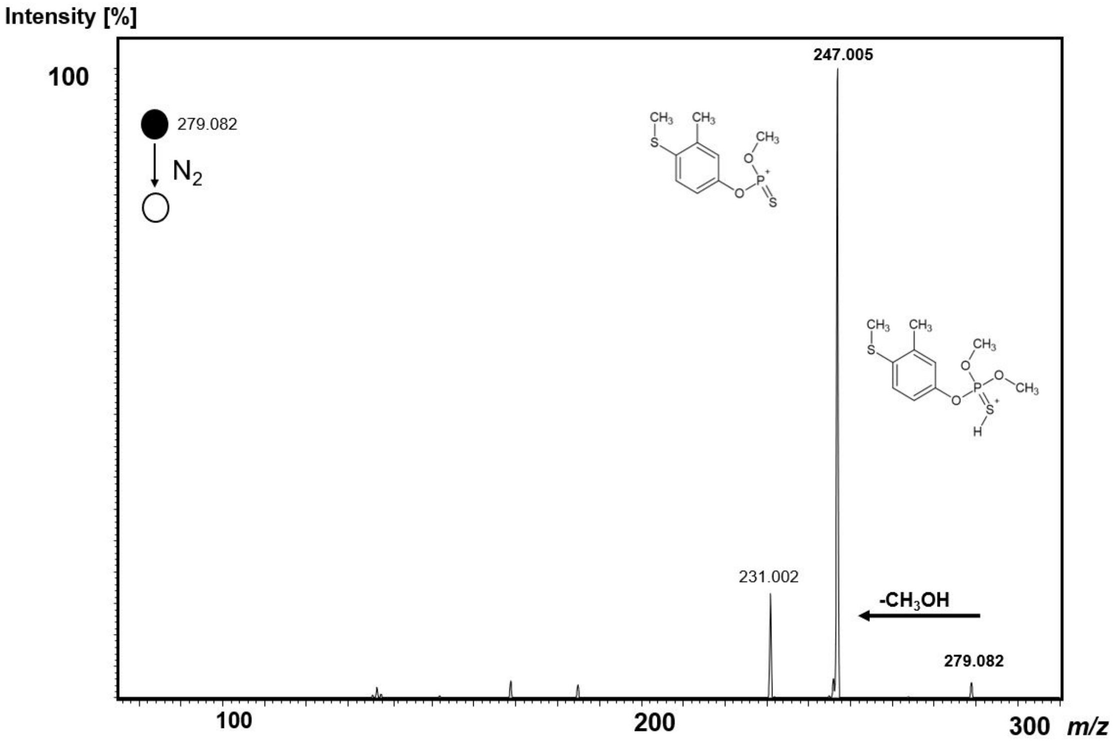

Figure S4: MS/MS spectra for fenthion (*m/z* 279.028)

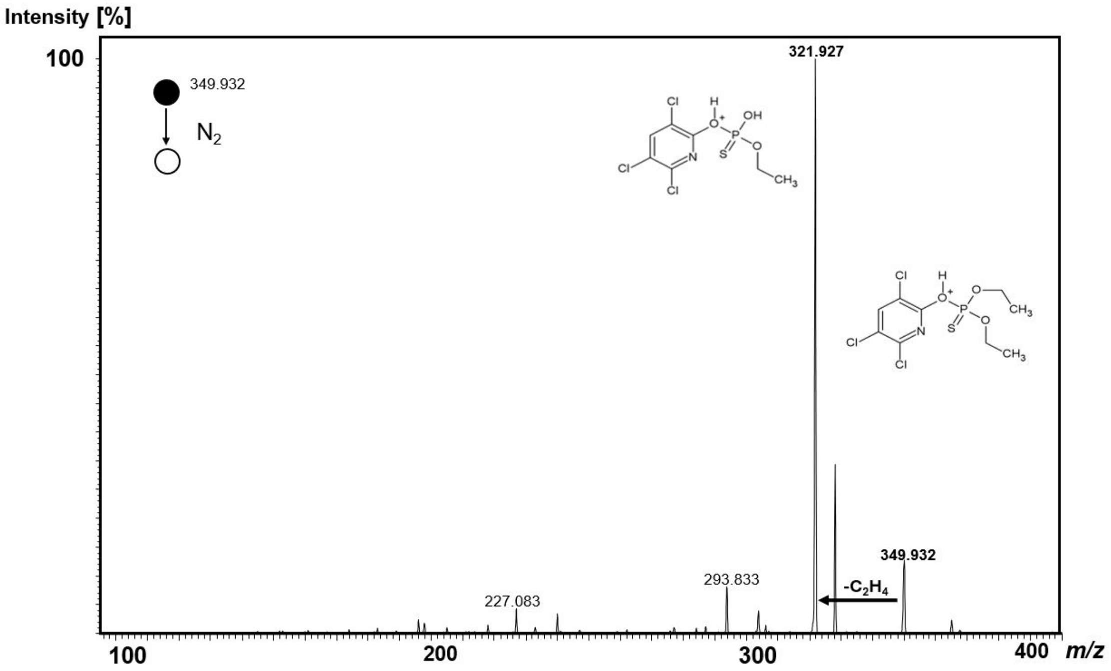

Figure S5: MS/MS spectra for chlorpyrifos *(m/z* 349.933)

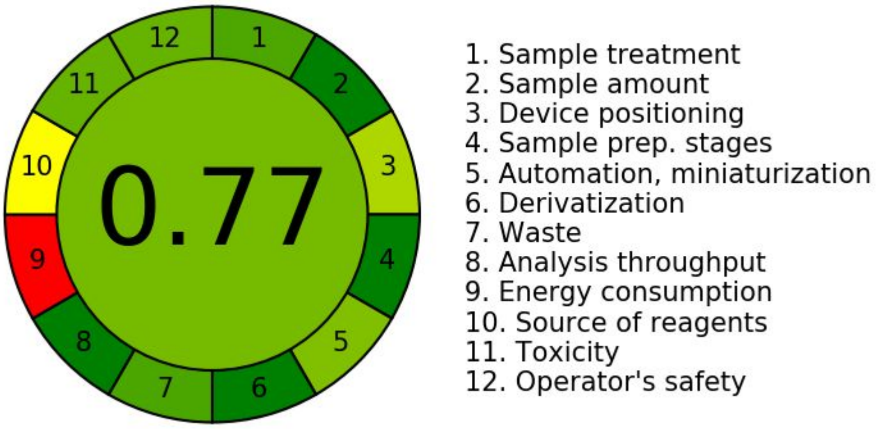

Figure S6. AGREE metric for the determination of antiparasitic compounds in cattle hair by DART-MS
